# Safety and preliminary efficacy of autologous bone marrow-derived mesenchymal stem cell transplantation in hereditary cerebellar ataxia: phase I/IIa clinical trial

**DOI:** 10.1186/s13287-026-05163-6

**Published:** 2026-07-10

**Authors:** Abbas Shapouri Moghaddam, Jalil Tavakol-Afshari, Najmeh Kaffash Farkhad, Majid Mojarrad, Maryam Eslami, Shahram Savad, Mohammad Ali Khodadoust, Ala Orafaie, Mohammadreza Sobhani, Akram Farmanbar, Mohammad Dashtkoohi, Hamid Khodayari, Delaram Hassani, Shahrzad Najafi, Parizad Najafi, Jürgen Hescheler, Karim Nayernia, Amirreza Boroumand

**Affiliations:** 1 Immunology Research Center, Department of Immunology, Faculty of Medicine, Mashhad University of Medical Sciences, Mashhad, Iran; 2 Department of Medical Genetics and Molecular Medicine, School of Medicine, Mashhad University of Medical Sciences, Mashhad, Iran; 3 Applied Biotechnology Research Center, Tehran Medical Sciences, Islamic Azad University, Tehran, Iran; 4 International Faculty, Tehran Medical Sciences, Islamic Azad University, Tehran, Iran; 5 Department of Genetics, Faculty of Advanced Science and Technology, Tehran Medical Sciences, Islamic Azad University, Tehran, Iran; 6 Department of Medical Genetics, School of Medicine, Tehran University of Medical Sciences, Tehran, Iran; 7 Endoscopic and Minimally Invasive Surgery Research Center, Ghaem Hospital, Mashhad University of Medical Sciences, Mashhad, Iran; 8 Department of Neurology, School of Medicine, Mashhad University of Medical Sciences, Mashhad, Iran; 9 International Center for Personalized Medicine (P7Medicine), 40235 Düsseldorf, Germany; 10 Neuroscience Research Center, Mashhad University of Medical Sciences, Mashhad, Iran; 11 European Center for Personalized Medicine, Medical Center, European Cell Medicine Institute, Düsseldorf, Germany

**Keywords:** Cerebellar ataxia, Mesenchymal stem cells, Clinical trial

## Abstract

**Background:**

Hereditary cerebellar ataxias (HCA) encompass a spectrum of pathological conditions affecting the cerebellum. Currently, there is growing interest in the potential role of mesenchymal stem cells (MSCs) as an investigational therapeutic approach for this condition. Hence, the objective of this single-center, open-label, phase I/IIa clinical trial was to assess the safety and exploratory clinical and biomarker changes following a single intrathecal injection of autologous bone marrow-derived mesenchymal stem cells (BM-MSCs) in HCA.

**Methods:**

Ten confirmed patients with HCA entered the study and underwent a single dose of (1 × 10^6^ cells/kg BW) intrathecal transplantation of BM-MSCs at passage 3. During the follow-up, patients were evaluated four times (one month before the intervention (-1), months 1, 3, and 6). Assessments included safety evaluation, Scale for the Assessment and Rating of Ataxia (SARA), GAD 65-antibody, and specific cytokines in the patient’s serum and cerebrospinal fluid (CSF) samples.

**Results:**

No severe adverse effects were observed following the cell transplantation procedure. A decreasing trend in SARA score was observed, with a statistically significant difference at month 6 compared with baseline. Except for one patient, GAD-65 antibody levels remained within the normal range in all patients. In one patient with markedly elevated baseline GAD-65 titers, serum and CSF GAD-65 levels decreased from 814 IU/mL and 781 IU/mL, respectively, to values within the normal range after 3 months. Significant changes were observed in selected inflammatory biomarkers, including decreased serum IL-6 at month 3, decreased CSF TNF-alpha at months 1 and 3, and increased serum IL-10 during follow-up. CSF IL-6 did not show a significant decrease.

**Conclusion:**

The findings support the short-term safety and tolerability of a single intrathecal dose of autologous BM-MSCs in this small HCA cohort. The study provides preliminary signals of possible clinical and biomarker changes; however, efficacy cannot be established due to the uncontrolled design, small sample size, disease heterogeneity, and short follow-up. Larger randomized controlled trials are required to validate these exploratory findings.

*Trial registration:* This clinical trial was registered with the Iranian Registry of Clinical Trials (ID: IRCT20160809029275N3).

## Introduction

 Hereditary cerebellar ataxia is a genetic disorder with a wide range of phenotypic and genotypic variations, characterized by progressive disturbances in gait and other movement functions. Friedreich ataxia (FRDA), is the most prevalent type with recessive inheritance. This disorder is associated with inordinate GAA repeats in the first intron of the frataxin (FXN) gene. In individuals with FRDA, the levels of mature frataxin protein are low. Frataxin plays a role in mitochondrial iron homeostasis. Low frataxin levels lead to an overproduction of free radicals, increasing sensitivity to oxidative stress and cell death [1, 2]. Numerous treatment approaches have been put forth to slow the progression of FRDA. Most of which seek to enhance antioxidant defence mechanisms, boost mitochondrial function, or elevate frataxin expression [3].

The most common types with dominant inheritance are spinocerebellar ataxia (SCA) types 1 and 2. SCAs typically have adult-onset disorders and include more than 30 clinically and genetically diverse neurological disorders. Currently, no treatments are available that can change the progression of the disease [4]. Chang et al.‘s study showed that the transplantation of human mesenchymal stem cells can delay the deterioration of the motor function of SCA2 transgenic mice. The migration of these cells to the white matter and cortex of the cerebellum caused the protection of Purkinje cells in these areas from the damage caused by the disease [5].

Researchers are exploring innovative therapeutic strategies. Among these, mesenchymal stem cell therapy has emerged as a promising approach for promoting neurological restoration and facilitating functional recovery in patients [6, 7]. Mesenchymal stem cells are multipotent stem cells found in bone marrow, adipose, and umbilical cord, with remarkable regenerative and immunomodulatory properties [8, 9]. These cells can differentiate into multiple cell lineages, including neurons and glial cells, and secrete a wide range of bioactive molecules that promote tissue repair, modulate inflammatory responses, and enhance neuroprotection. Additionally, MSCs can efficiently inhibit apoptosis in neurons and glial cells, decrease oxidative damage, further contributing to their therapeutic potential [1, 5, 10, 11]. Their exceptional in vitro expansion capability also facilitates the rapid attainment of the required cell quantity for in vivo therapy [12]. These characteristics position MSCs as an attractive candidate for therapeutic intervention in HCA, offering the potential to halt or reverse disease progression.

Encouraging results have been reported in preclinical studies using animal models, demonstrating that MSC transplantation can improve motor coordination, restore cerebellar circuitry, decrease apoptotic markers, modulate inflammatory responses and enhance neuronal survival [13–18]. Although HCAs are genetically heterogeneous, accumulating evidence suggests that several common downstream mechanisms contribute to cerebellar neuronal loss and dysfunction. These include mitochondrial impairment, oxidative stress, excitotoxicity, and secondary inflammatory responses [19, 20]. Mesenchymal stem cells have been shown to exert multiple neuroprotective and trophic effects through paracrine secretion of growth factors such as brain-derived neurotrophic factor (BDNF), glial cell line-derived neurotrophic factor (GDNF), and vascular endothelial growth factor (VEGF) [21]. In addition, MSC modulate microglial activation, attenuate oxidative stress, and promote neuronal survival and synaptic repair. Therefore, despite the genetic heterogeneity of HCAs, MSC therapy may target these shared downstream pathogenic pathways and confer broad neuroprotective benefits [15, 22]. These preclinical findings have paved the way for clinical trials evaluating the safety and potential efficacy of MSC therapy in patients with hereditary cerebellar ataxia (HCA). Preliminary clinical data from early-phase, mostly uncontrolled studies have demonstrated favorable safety profiles together with signals of potential benefit, including improvements in motor function and quality of life [23, 24]. However, similar to the present study, these trials remain exploratory and do not constitute definitive proof of clinical efficacy, owing to their small sample sizes, lack of control groups, and participant heterogeneity [25]. Accordingly, the objective of this study was to evaluate the safety and preliminary efficacy of autologous bone marrow-derived mesenchymal stem cells (BM-MSCs) in patients with HCA.

## Materials and methods

### Study design

This study is a single-site, single-arm, open-label, phase I/IIa clinical trial conducted at Pastoor Hospital, Mashhad University of Medical Sciences, and Mashhad, Iran. The study was performed in accordance with the principles of the Declaration of Helsinki and approved by the Ethics Committee of Mashhad University of Medical Sciences (IR.MUMS.REC.1401.128). It was also registered in the Iranian Registry of Clinical Trials (IRCT20160809029275N3). Although the original registered protocol included a comparison group and several additional outcome measures, the present study represents a protocol deviation due to funding limitations and restrictions related to international sanctions, which prevented completion of all planned assessments. Consequently, the current report includes all data that were successfully collected and analyzed, while acknowledging that these deviations limit interpretability, increase the risk of bias (including inability to control for natural history or placebo effects), and reduce generalizability. These limitations further highlight the practical challenges of conducting clinical trials in resource-constrained settings and emphasize the need for adequately powered, controlled studies to more rigorously evaluate the safety and efficacy of this therapeutic approach.

All participants provided informed consent to take part in the trial. The privacy rights of patients in this study were upheld. In this study, the term “sex” refers to biological distinctions based on the assigned and documented sex at birth.

### Participant eligibility

Ten patients were enrolled in this trial and received the intervention. All patients had a confirmed diagnosis of HCA by a neurologist based on MRI scans of the brain and cervical spinal cord and genetic tests. During the study, all patients continued their standard medical care throughout the trial period. There were no specific interventions to alter or intensify rehabilitation or other standard therapies during participation. This approach was intended to maintain stability in concomitant treatments and minimize potential confounding effects on functional outcomes. While intensive rehabilitation could theoretically influence results, the study design did not include changes to standard therapy protocols, thereby reducing the likelihood that such factors confounded the observed outcomes. The inclusion and exclusion criteria were as follows:

#### Inclusion criteria

- Patients between 10 and 65 years old without sex limitation.
- Signing an informed written consent.
- Definitive diagnosis of HCA based on brain and cervical spinal cord MRI and/or genetic test.
- Presence of cerebellar or brainstem atrophy and/or cervical spinal cord.
- Not having other mental illnesses such as schizophrenia, and/or structural brain disorders.

#### Exclusion criteria

- Positive pregnancy test.
- Heart, kidney, and liver failure, such as cardiac arrhythmia, diabetes, leukemia, epilepsy, lung disease, and other diseases of the central nervous system such as Parkinson’s.
- Having a total bilirubin level exceeding 1.5 times the upper limit of its normal value.
- History of chronic or acute alcohol consumption.
- Any evidence of infection.
- Failure to complete a trial.
- Severe psychosis, cognitive disorders, and inability to understand or sign consent.
- Other severe organic or systemic diseases.
- Potential long-term effects of previous trials that may bias the results.
- Presence of thyroid disease.
- Acute causes of ataxia.

**Fig. 1:**
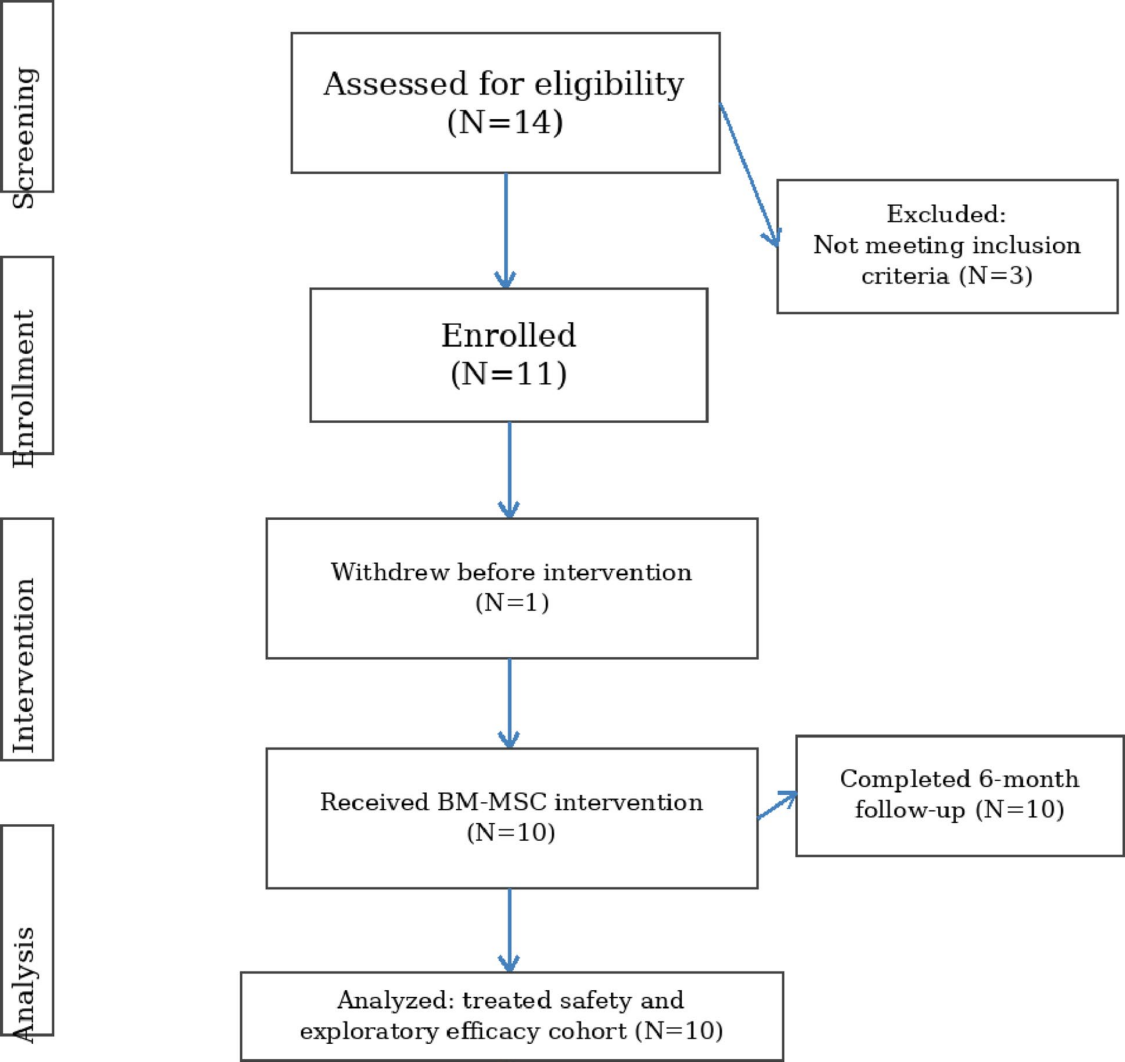
Participant flow diagram for this single-arm, open-label, non-randomized Phase I/IIa clinical trial. (adapted from CONSORT 2010 guidelines for transparent reporting of non-randomized studies). Fourteen patients were screened, 11 were enrolled, and 10 received the intervention and completed the 6-month follow-up

A total of 14 patients were screened for eligibility. Eleven patients met all inclusion/exclusion criteria and provided informed consent, thus constituting the enrolled cohort (*N* = 11). Of the 11 enrolled patients, one participant withdrew consent before receiving the intervention (intrathecal injection of BM-MSCs). The reason for withdrawal was reported as dissatisfaction with the study protocol/logistics. The remaining ten patients (*N* = 10) received the scheduled intrathecal injection of autologous BM-MSCs. All ten treated participants completed the full 6-month follow-up period and were included in the final safety and exploratory efficacy analyses. Because one participant withdrew before receiving the intervention, analyses were conducted on the treated cohort rather than a formal intention-to-treat population. This process is summarized in Fig. 1, which presents the participant flow for this single-arm, open-label, non-randomized Phase I/IIa clinical trial.

### Intervention and follow-up period

Injections were administered in a clinical setting, following the standard lumbar puncture procedure at the L3-L4 level over 2 min. Each injection contained 1 × 10^6^ autologous bone marrow-derived mesenchymal stem cells per kilogram of body weight [23, 26] .The total volume of each solution was increased to 2 ml by adding normal saline. The follow-up period lasted six months, with evaluations at 1-, 3-, and 6-months post-intervention. Additionally, all patients were evaluated one month prior to the intervention. In all stages of the study, patients received standard care in addition to the BM-MSC therapy.

### Bone marrow extraction

The bone marrow extraction procedure was performed on the patient’s posterior superior iliac crest. The patient was positioned in either a left or right lateral decubitus stance and received local anesthesia to ensure comfort during the procedure. A single aspiration was performed, extracting around 50 ml of bone marrow blood from each patient. The puncture site was evaluated on the following day, and if no complications were detected, the patient was discharged from the medical facility.

### BM-MSCs preparation, characterization, and transplantation

Isolation and preparation of cells were conducted according to our previous protocol [27], briefly described as follows: The Ficoll preparation method (using Ficoll from GE Healthcare Bio-Sciences in Uppsala, Sweden) was applied for bone marrow mononuclear cell separation. The isolated mononuclear cells, totaling 3 × 10^6^ cells, were cultured in a 175-cm^2^ flask (Thermo Scientific Nunc in Roskilde, Denmark). The culture medium consisted of enriched minimum essential medium-alpha (α-MEM, Biowest, South America) supplemented with 10% fetal bovine serum (FBS, Life Technologies in Grand Island, USA), and 1% penicillin-streptomycin (Gibco000, USA). The cells were incubated in a humidified incubator at 37 °C with 5% CO2. To remove non-adherent cells, the culture medium was replaced, and this process was repeated twice a week. Upon reaching 80% confluence, the primary cultures of BM-MSCs were harvested using 0.125% trypsin-EDTA (Gibco, USA), and passaged.

**Fig. 2:**
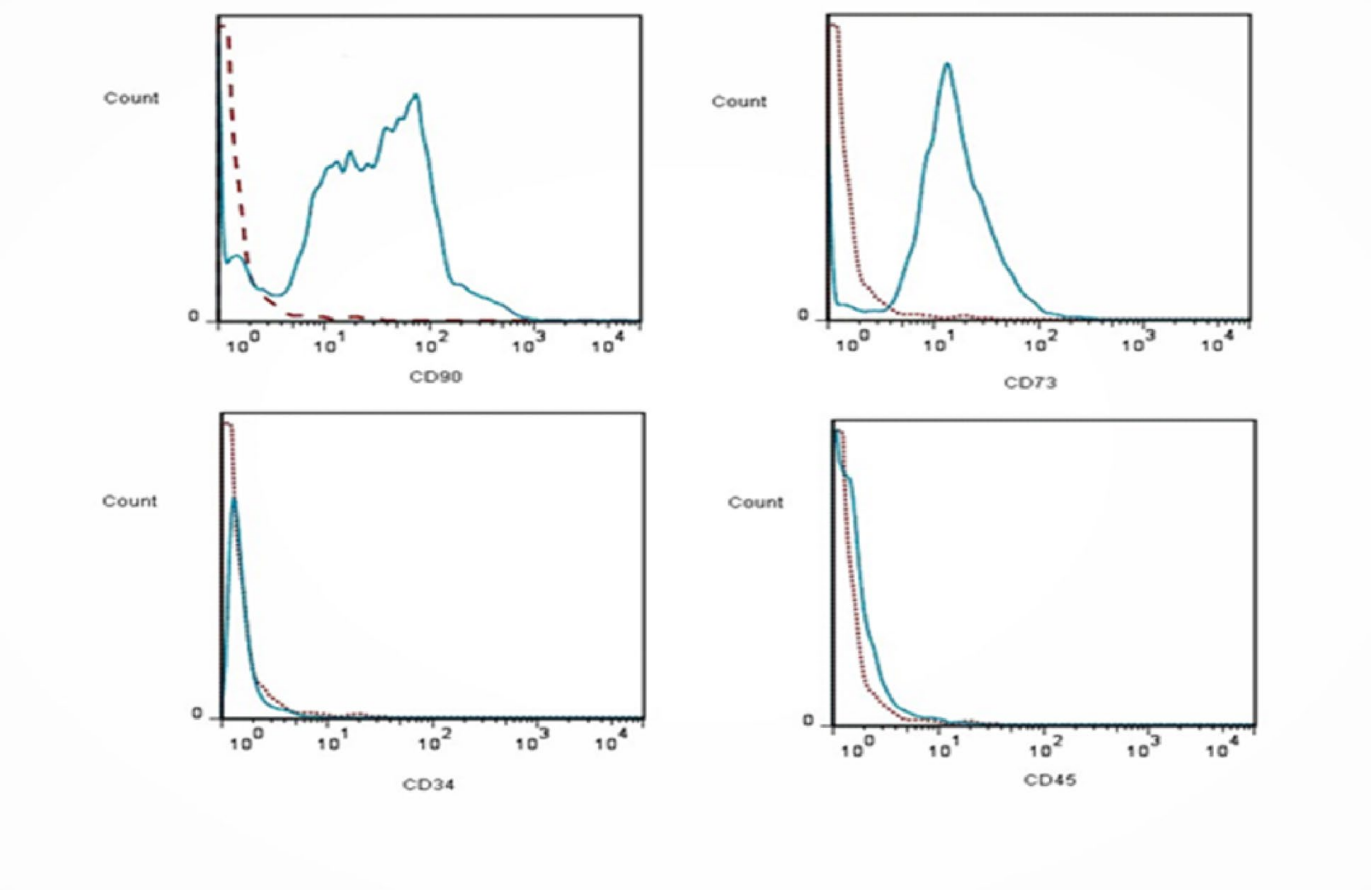
Immunophenotypic characterization of human Bone marrow-derived Mesenchymal Stromal cells by flow cytometry for the expression of mesenchymal (CD73, CD90) and hematopoietic (CD34, CD 45) stem cells markers. (Dotted line: unstained control, Solid line: a marker of interest)

**Fig. 3:**
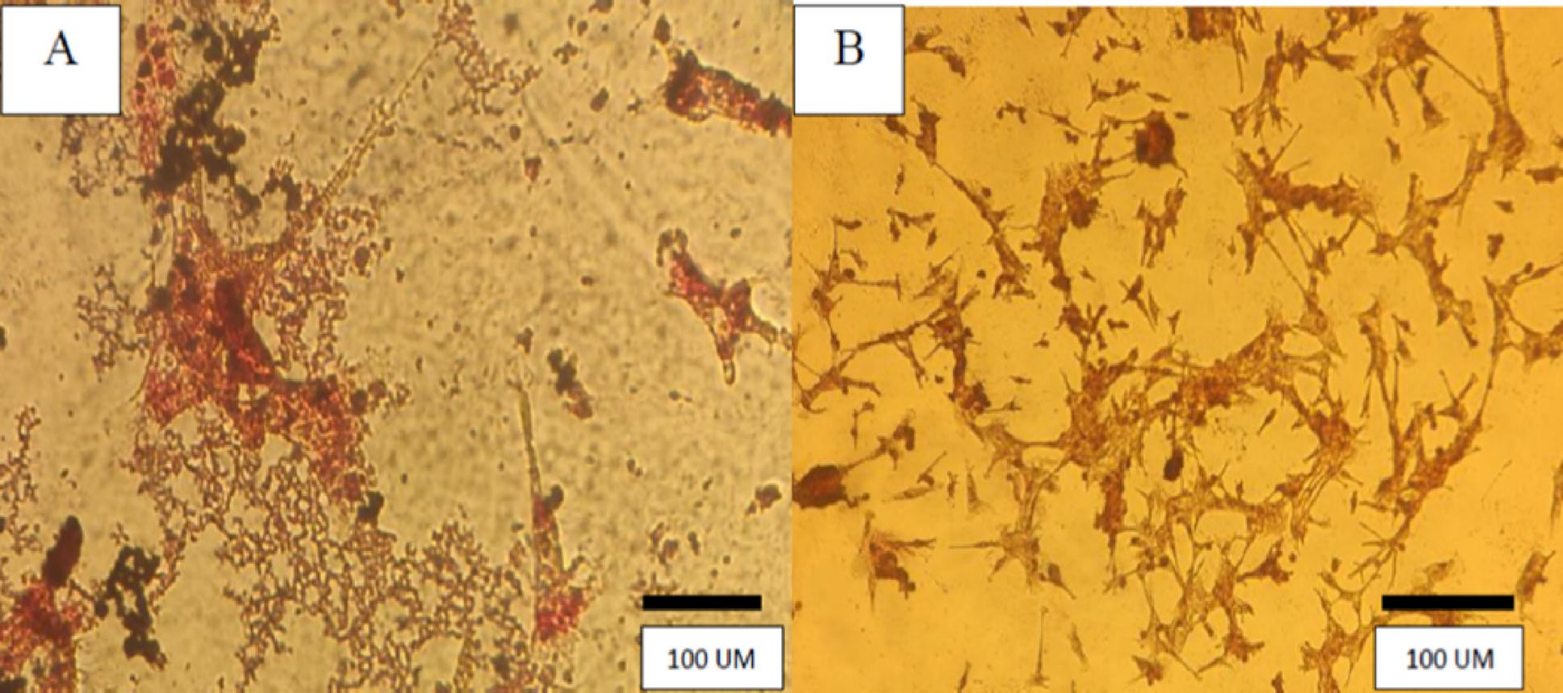
**A** Adipogenic and **B** osteogenic differentiation of BM-MSCs (× 100 from Inverted phase microscope). BM-MSCs: Bone Marrow-derived Mesenchymal Stem Cells

Subsequently, the characterization of BM-MSCs at the third passage (P3) was conducted according to the guidelines set by the International Society for Cellular Therapy (ISCT). This characterization involved flow cytometry analysis, as depicted in (Fig. 2). Furthermore, the differentiation potential of BM-MSCs into adipocytes (Fig. 3A) and osteocytes (Fig. 3B) was evaluated following our previously established protocols [28, 29]. Specifically, the osteogenic differentiation of MSCs was assessed using Alizarin Red S staining obtained from Kia Zist, Iran. Similarly, Oil Red O staining from the same source was employed to examine the adipogenic lineage differentiation capacity of MSCs.

To ensure sterility, the samples underwent rigorous testing for bacteria, mycoplasma, and yeast or fungi using endotoxin tests, polymerase chain reaction (PCR), and Bactec tests, respectively. The viability of the cells prior to transplantation ranged between 95% and 98%. Finally, the autologous BM-MSCs product at the third passage (P3) was released and transported under controlled temperature conditions (2–8 °C) to the investigation site. After preparation and before injection, the cells were washed 3 times with injectable sterile normal saline to remove materials related to the culture medium or FBS.

Cells were expanded in medium containing 10% FBS and washed extensively (three times) with sterile normal saline prior to administration. Although rigorous sterility, viability, and endotoxin testing was performed, the use of xeno-derived FBS remains a theoretical concern regarding residual bovine proteins in contemporary Good Manufacturing Practice (GMP) and Advanced Therapy Medicinal Product (ATMP) standards. This is acknowledged as a limitation of the present study, and xeno-free culture systems are preferred for future clinical applications to minimize potential immunogenic and regulatory risks [30, 31].

All the aforementioned procedures, including BM-MSC harvesting, processing, and product release, were carried out in a Grade B cleanroom that met the requirements of GMP.

$\:{10}^{6}$At the investigation site, the BM-MSCs were delivered intrathecally (IT) at the L3-L4 level using a standard lumbar puncture technique. The cell suspension consisted of 1 × 10^6 cells per kilogram of body weight, formulated in 2 mL of normal saline solution, and was injected slowly over an approximate two-minute duration.

**Fig. 4:**
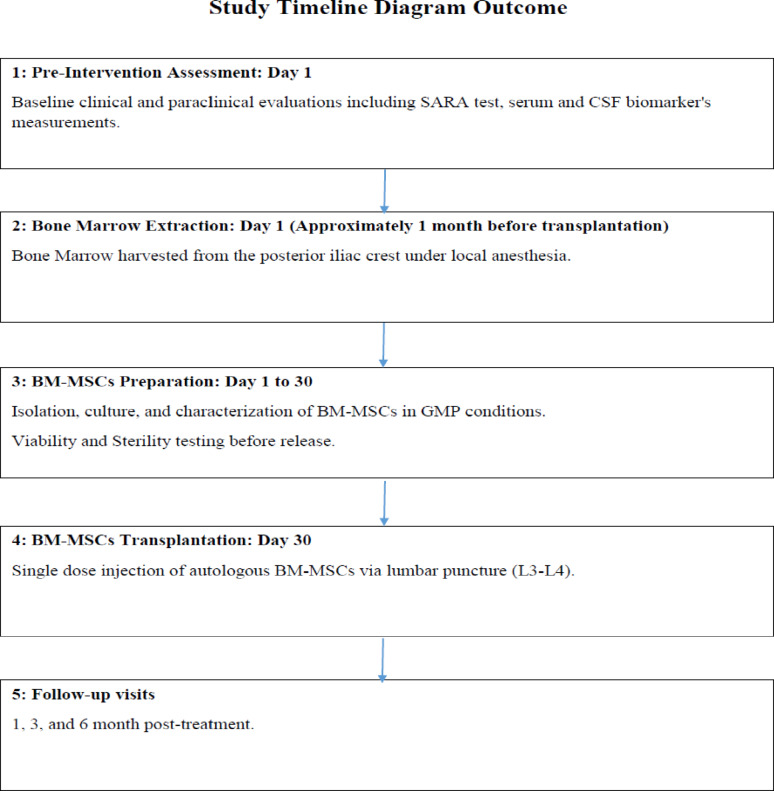
Study timeline diagram outcome

Figure 4 illustrates the approximate timing of key procedures and follow-up assessments, including baseline evaluation, bone marrow extraction, BM-MSC preparation and release testing, intrathecal transplantation, and post-treatment visits at months 1, 3, and 6.

### Endpoints

#### Primary outcome

The primary objective of this study was to investigate the safety of single doses injection of autologous BM-MSC in hereditary CA patients. To ensure safety, a clinician closely monitored the patients for 24 to 48 h after injection, specifically looking for any possible side effects like fever, respiratory disorder or potential allergic reactions ranging from skin lesions to severe anaphylactic reactions. If any severe symptoms were observed during the monitoring period, the patient was immediately excluded from the study and underwent the supplementary therapy. The levels of serum CRP, as well as liver enzymes (ALT & AST) were also analyzed during the pre- and post-treatment for safety monitoring.

#### Secondary outcome

The secondary aim of this study was to evaluate exploratory clinical and biomarker changes following the intervention in affected patients. To investigate the effects of BM-MSCs, the study examined various parameters both in serum and CSF of patients, including GAD-65 antibodies, IL-6, TNF-alpha, and IL-10 levels using ELISA analysis (ELISA, Karmania Pars gene, Iran). These measurements were taken four times: one month before intervention, and at months 1, 3, and 6 after intervention. SARA scores were also evaluated at these time points. The SARA test is a reliable and valuable tool for evaluating individuals with cerebellar ataxia during routine assessments, as it correlates with disease severity [32].

Due to lack of sufficient funding and restrictions related to international sanctions, several of the additional assessments planned in the trial registry could not be conducted; therefore, the present report includes all data that were successfully collected and analyzed.

### Data collection

This study took place at Pastoor Hospital, Mashhad University of Medical Sciences, located in Mashhad, Iran, spanning from September 2021 to Jun 2022. During this period, the research team collected and documented all relevant clinical and paraclinical data from the patients’ records.

### Statistical analysis

As this study was a phase I/II clinical trial, the sample size was determined based on previous limited studies as well as the availability of referring patients. No special formula was used to determine the sample size.

To assess the normality of the distribution, the Shapiro-Wilk test was applied. The impact of time on normally distributed data was analyzed using generalized linear models (GLM) and repeated measures analysis of variance (RM ANOVA). In the case of non-normally distributed continuous variables, the results were reported as the median along with the interquartile range (IQR). A comparison of medians between two related groups was performed using either paired t-tests or Wilcoxon signed-rank tests, as appropriate. Continuous variables were presented as either the mean (for normally distributed data) or the median (for non-normally distributed data) ± the standard error of the mean/median (SEM). Paired-sample t-tests were used to evaluate pre- and post-transplantation paraclinical parameters when appropriate. The statistical analysis was carried out using IBM SPSS version 23.0, and statistical significance was determined at a level of *P* < 0.05. Given the exploratory nature of this phase I/IIa study and the small sample size, no adjustment for multiple comparisons was performed; therefore, secondary efficacy and biomarker findings should be interpreted as hypothesis-generating.

## Results

### Clinical characterization of patients and safety assessment

In this clinical trial, 10 treated patients were included in the final safety and exploratory efficacy analyses (7 male and 3 female). Eight patients had Friedreich’s ataxia (FRDA) and two patients had spinocerebellar ataxia (SCA1 and SCA2). Due to the small number of SCA cases, results are presented in aggregate form (see Discussion for further details on disease heterogeneity). The average age of the patients was 32 years. Table 1 briefly shows the demographic and clinical details of the patients at baseline. Regarding procedural safety, none of the treated patients experienced severe complications related to the injection. Patients P3, P6, and P8 had mild headaches after the injection. Headaches responded to 500 mg acetaminophen and resolved within 24 h. P7 developed a low-grade fever without any other complication, which also responded to acetaminophen and resolved. There were no reports of severe complications in any treated patient during follow-up. One enrolled participant withdrew consent before receiving BM-MSCs and was therefore not included in the treated safety or exploratory efficacy cohort. All ten treated participants completed the 6-month follow-up.

Also, there was no significant change in the amount of liver enzymes and CRP levels before and after transplantation (Table 2).

**Table 1:** Demographic data and clinical characterization of finally included patients at baseline

Patient Number	Age at onset (years)	Diagnosis	Sex	Disease duration (years)	Baseline SARA score
P1	41	FRDA	Male	9	2
P2	43	FRDA	Male	2	5
P3	21	FRDA	Female	9	12
P4	37	SCA2	Male	11	22
P5	18	FRDA	Female	13	21
P6	38	FRDA	Female	10	18
P7	21	SCA1	Male	12	18
P8	42	FRDA	Male	5	5
P9	37	FRDA	Male	10	14
P10	24	FRDA	Male	6	18

FRDA: Friedreich’s Ataxia, SCA: Spinocerebellar ataxia

**Table 2:** Paraclinical parameter’s evaluation for safety monitoring

Paraclinical parameters	CRP (mg/L)		ALT(IU/L)		AST(IU/L)	
	Before	After	Before	After	Before	After
Mean ± SEM	2.86 ± 0.38	2.23 ± 0.28	26.2 ± 17.84	22.7 ± 13.58	23.1 ± 7.24	22.2 ± 7.83
P.value	0.84		0.48		0.06	

CRP: C - reactive protein/ ALT: Alanine aminotransferase/ AST: Aspartate aminotransferase

### Efficacy assessments

#### SARA score

**Fig. 5:**
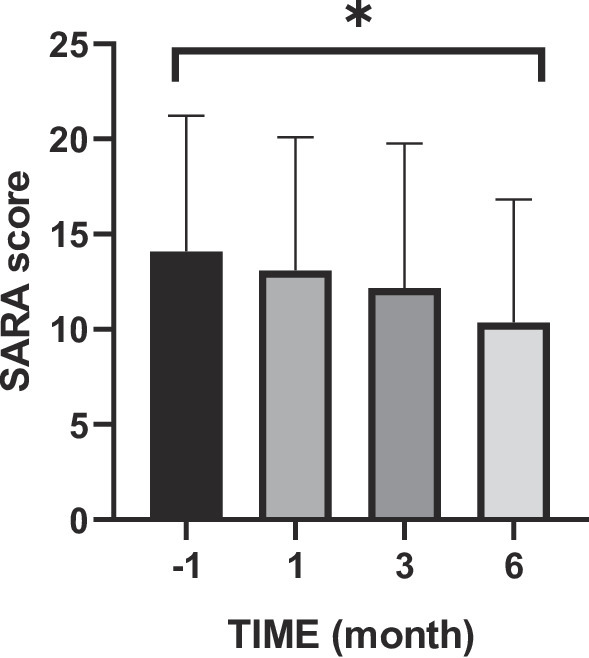
SARA (Scale for the Assessment and Rating of Ataxia) score of patients during the study. *P < 0.05

We evaluated the SARA score in all patients one month before intervention and in the follow-ups. SARA score was significantly lower in the sixth month (10.36 ± 1.94) compared to baseline (month − 1) (14.9 ± 2.1, *P* = 0.002). (Fig. 5) illustrates the SARA scores at baseline and one, three, and six months following the administration of BM-MSCs treatment.

#### Anti-GAD levels

**Fig. 6:**
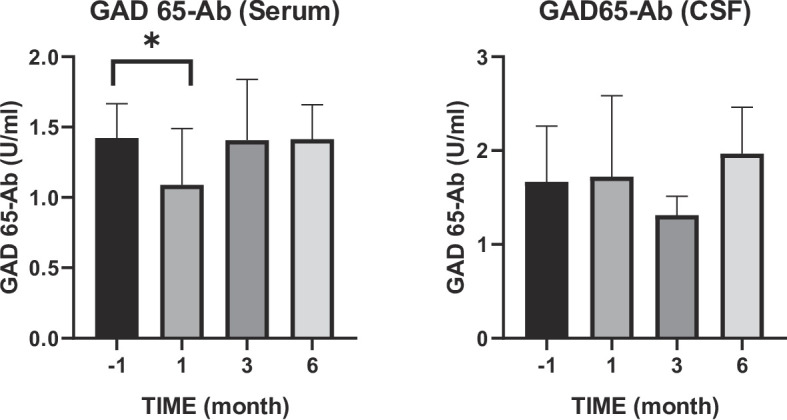
**A** Serum GAD-65 levels **B** GAD-65 levels in CSF. *P < 0.05

As depicted in Fig. 6A, the serum levels of GAD-65 remained consistently within the normal range (< 5 IU/ml) across all four measurement periods in most patients. Although a significant decrease within the normal range was observed at the first month (1.08 ± 0.13) compared with the pre-intervention measurement (1.42 ± 0.08), subsequent assessments after cell therapy showed only a non-significant further decline relative to baseline values. One patient (P2), however, deviated markedly from this trend. This patient demonstrated an initial serum GAD-65 level of 814 IU/ml, which subsequently declined to 748, 1.7, and finally 1.7 IU/ml across the following three measurements. Notably, after three and six months of cell therapy, the antibody levels had decreased dramatically and returned to the normal range. Regarding CSF GAD-65 levels during the study period, no significant changes were observed compared with baseline (month − 1), and values remained within the normal range (< 2 IU/ml) throughout the study in all patients except P2. In this patient, CSF GAD-65 levels followed a pattern similar to that observed in serum, declining from 781 to 678, 1.3, and 1.8 IU/ml over the course of the study. Importantly, after three and six months of stem cell therapy, CSF antibody levels also decreased substantially and normalized (Fig. 6B). While the marked reduction in GAD-65 levels observed in this single patient, together with the cytokine profile changes (decreased IL-6/TNF-α and increased IL-10), is noteworthy and aligns with the known immunomodulatory properties of MSCs, these findings should be interpreted cautiously because of the small sample size, disease heterogeneity, and the largely normal baseline inflammatory status characteristic of hereditary (non-primary autoimmune) ataxias. These observations may support the possibility of systemic immunomodulatory effects following MSC therapy; however, they do not establish a definitive CNS-localized mechanism or a direct causal relationship with the observed clinical changes, and potential assay variability as well as natural biological fluctuations must also be taken into consideration, such that the present findings should be regarded as exploratory and hypothesis-generating rather than conclusive evidence of efficacy or mechanism [33].

#### Cytokine analysis

##### IL-6 levels

**Fig. 7:**
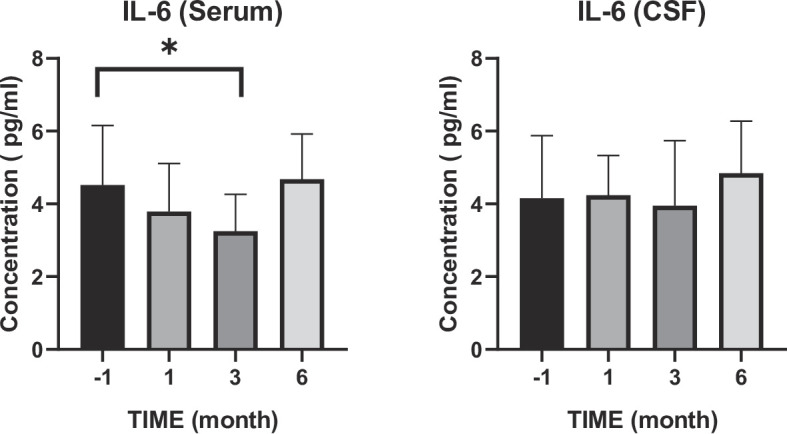
**A** Serum IL-6 levels** B** IL-6 levels in CSF. *P < 0.05

Figure 7 A Demonstrates a notable decline in serum levels of IL-6 cytokines following BM-MSCs transplantation during the initial three months of study. Specifically, the decrease observed in the third month (3.25 ± 0.41) compared to the 1 month before transplantation (4.51 ± 0.51) was statistically significant (*P* = 0.03). While there is a subsequent increase in this cytokine during the sixth month (4.57 ± 0.39), it was not statistically significant (*P* = 0.83).

Also, the amount of this cytokine in the patients’ CSF showed a slight decrease during the study period. However, an increase was observed in month 6 (4.84 ± 0.45) compared to month − 1 (4.15 ± 0.54), which was not statistically significant (*P* = 0.27) (Fig. 7B).

##### TNF-α levels

**Fig. 8:**
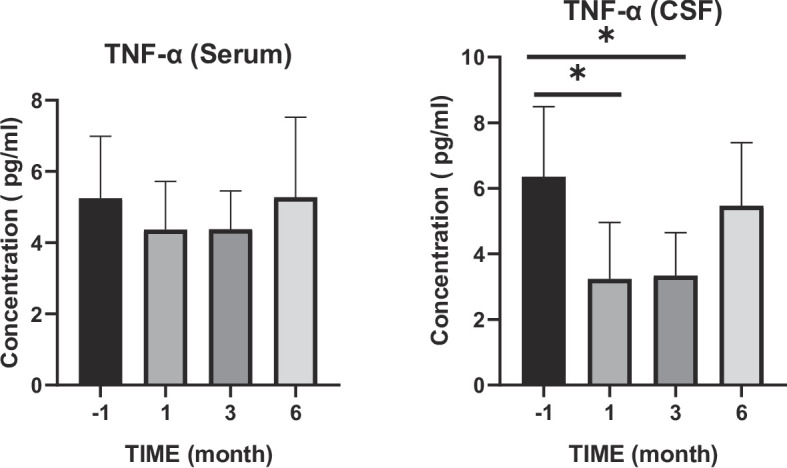
**A** Serum TNF-alpha levels** B** TNF-alpha levels in CSF. *: *P* < 0.05

As shown in (Fig. 8A) The serum levels of TNF-α cytokine exhibited a declining pattern during the initial three months and returned to baseline levels by the sixth month. However, none of these changes were found to be statistically significant when compared to the pre-intervention levels. Besides, the concentration of this cytokine in the patients’ CSF demonstrated a declining trend throughout the study period. Specifically, the decrease in the first (3.23 ± 0.54, *P* = 0.002) and third months (3.34 ± 0.41, *P* = 0.002) compared to the pre-intervention month (6.35 ± 0.67) exhibited statistically significant changes (Fig. 8B).

##### IL-10 levels

**Fig. 9:**
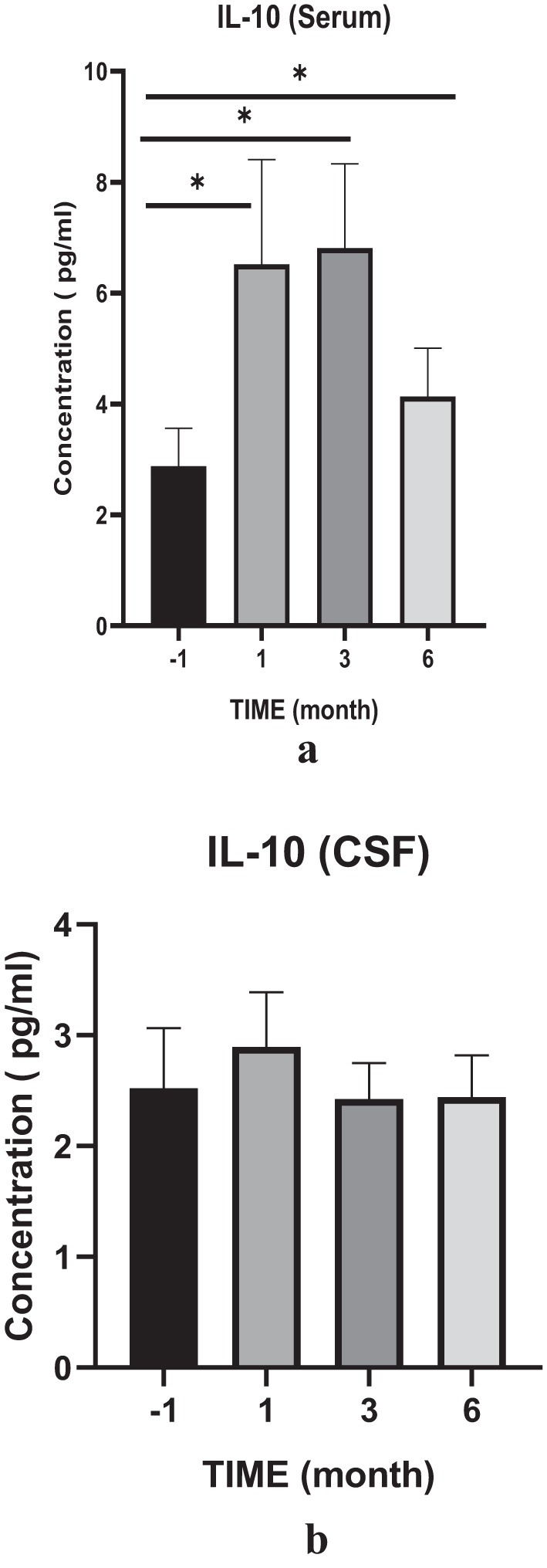
**A** Serum IL-10 levels ** B** IL-10 levels in CSF. *: *P* < 0.05

According to (Fig. 9A), the serum levels of IL-10 cytokine displayed a notable increase in months 1 (6.51 ± 0.59, *P* < 0.0001), 3 (6.81 ± 0.48, *P* < 0.0001), and 6 (4.63 ± 0.25, *P* = 0.002) in comparison to the pre-transplantation month (2.87 ± 0.21). However, there was no statistically significant change observed in the amount of this cytokine in the patients’ CSF throughout the transplantation period (Fig. 9B).

## Discussion

### Safety

The findings of this clinical trial support the short-term safety and tolerability of intrathecal autologous BM-MSC administration in this small cohort of patients with HCA, with exploratory signals of clinical change. No severe adverse effects were detected during administration or follow-up. Mild complications included headache and low-grade fever, which were promptly alleviated with appropriate medication.

A recent systematic analysis of nine clinical trial studies has demonstrated the safety and tolerability of intrathecal stem cell transplantation for neurological disorders [34]. Furthermore, the outcomes of a one-year clinical trial involving seven patients with SCA-3 and MSA-C have indicated that intravenous transplantation of allogeneic adipose mesenchymal stem cells (1 × 10^6^ cells/kg BW) was safe and well-tolerated. Similarly, they reported complications irrelevant to the infusion of allogeneic MSCs. Additionally, vital signs, liver or kidney functions, or blood cell counts were unchanged [23]. In a case series study with three patients with FRDA, human embryonic mesenchymal stem cell transplantation was reported to be safe and well-tolerable, and they also reported physical improvement effects following cell transplantation [6].

In a clinical trial study, sixteen genomically diagnosed patients with SCA underwent intravenous and intrathecal infusions of umbilical cord MSCs (UC-MSCs). Clinical, laboratory, and imaging assessments were performed to evaluate safety. Over the 12-month follow-up, no severe adverse events related to the transplantation were reported. In another clinical trial, Dongmei et al. examined 25 patients, comprising 14 cases of SCA and 10 cases of multiple system atrophy with cerebellar features. They injected 1 × 10^6 cells/kg intrathecal UC-MSCs weekly for one month. The treatment was reported to be safe and was associated with possible clinical benefit, although the uncontrolled design limits definitive efficacy conclusions [26].

### Preliminary efficacy

The cumulative experience with MSCs in neurological disorders now includes several hundred patients across early-phase studies. Although this growing body of evidence increases confidence regarding safety and potential biological activity, the convergence of findings from uncontrolled studies should not be interpreted as proof of definitive efficacy. Accordingly, the present study should be considered hypothesis-generating and supportive of the rationale for future controlled trials [35].

The study population consisted predominantly of patients with FRDA (*n* = 8), with only a small number of SCA cases (*n* = 2). Because FRDA and SCAs differ substantially in genetic background, molecular pathophysiology, disease progression, and potentially therapeutic responsiveness, this study was not powered to evaluate subtype-specific effects. Therefore, outcomes are presented in aggregate form, and extrapolation beyond this cohort should be undertaken cautiously. For transparency, descriptive individual SARA trajectories are presented in (Fig. 5). Throughout the study period, a downward trend in SARA scores was observed, reaching statistical significance at month 6 compared with the pre-injection assessment. However, interpretation of SARA outcomes requires careful distinction between statistical significance and clinical significance, as statistically significant changes do not necessarily correspond to meaningful functional improvement. In this context, clinically meaningful benefit is often considered to reflect a 3–4 point change in SARA score associated with improvements in daily functioning and quality of life. Furthermore, we acknowledge the possibility of ceiling effects, particularly in patients with mild ataxia, as illustrated by Participant 1’s baseline SARA score of 2, where low initial scores inherently limit the ability to detect measurable improvement; consequently, future studies should incorporate complementary outcome measures capable of more sensitively detecting subtle clinical changes in individuals with lower disease severity.

In hereditary ataxias, the primary pathology is a genetic defect leading to protein dysfunction and subsequent neurodegeneration [36]. The resulting inflammation is a secondary neuroinflammatory response characterized by the activation of local glial cells (microglia and astrocytes) and the release of pro-inflammatory cytokines within the cerebellum. These localized CNS changes often do not produce a robust enough signal to elevate peripheral, systemic markers measured in the blood (e.g., CRP). The finding of normal systemic inflammatory markers, combined with the genetic confirmation of the hereditary ataxia, supports the primary diagnosis and rules out a highly active, acute ACA [37].

The concentrations of GAD-65 antibodies in the serum and CSF of most patients remained within the normal range and did not show a significant increase, except in one patient with markedly elevated baseline titers. Glutamic acid decarboxylase (GAD), a predominantly intracellular enzyme found in neurons and insulin-secreting pancreatic beta cells, exists in two isoforms known as GAD65 and GAD67. Its primary physiological role involves the decarboxylation of glutamate to gamma-aminobutyric acid (GABA), the principal inhibitory neurotransmitter in the central nervous system. GAD antibodies are linked to various neurological disorders, including stiff-person syndrome, chronic cerebellar ataxia, and limbic encephalitis, all thought to stem from impaired GABAergic transmission [38–40]. Elevated levels of this antibody are observed in chronic cerebellar ataxia, indicating disease progression. Cytokines and autoantibodies influence the pathogenesis of various autoimmune diseases [41]. We acknowledge the measurement of GAD-65 antibodies in a cohort primarily diagnosed with hereditary ataxia. While most patients did not show elevated titers, the marked reduction in GAD-65 levels in one patient with high baseline serum and CSF values is noteworthy. This single-patient observation may be compatible with a possible immunomodulatory effect of MSCs; however, it should be interpreted cautiously and cannot establish causality or a definitive treatment effect. Previous studies have indicated that individuals with ataxia may show altered levels of pro-inflammatory cytokines such as IL-6 and TNF-alpha [42, 43]. Cytokines associated with the immune system, including TNF-alpha, interleukin-1beta (IL-1beta), and IL-6, are modulators of functional communication between neurons and glial cells [44–46]. In the present study, selected inflammatory biomarkers showed exploratory changes, including a significant decrease in serum IL-6 at month 3, a significant decrease in CSF TNF-alpha at months 1 and 3, and a significant increase in serum IL-10 during follow-up. In contrast, CSF IL-6 did not show a significant decrease. These biomarker findings should therefore be viewed as exploratory and hypothesis-generating. Our hypothesis was not based on the premise that transplanted autologous MSCs differentiate into new, healthy neurons or Purkinje cells to replace those lost due to the genetic defect; instead, the rationale is based on paracrine and neuroprotective mechanisms of MSCs. The observation that baselines inflammatory markers were largely within normal limits is consistent with the non-primary inflammatory nature of hereditary ataxias, although modest modulation after MSC infusion may still reflect systemic immunomodulatory effects rather than correction of an overt inflammatory state. Similarly, the use of autologous BM-MSCs in genetic disorders such as FRDA/SCA relies primarily on paracrine/immunomodulatory mechanisms rather than direct replacement of defective cells; however, MSC quality and regenerative potential may decline with donor age, affecting proliferation, differentiation, and secretome. Although the oldest patient in our cohort was 43 years, autologous harvesting from older individuals (> 50–60 years) can be technically challenging due to limited marrow volumes. Therefore, allogeneic MSCs from young healthy donors, such as Wharton’s jelly umbilical cord-derived MSCs, may offer advantages in consistency and potency for future studies [47, 48].

Preclinical and clinical investigations have demonstrated temporary positive effects of stem cell-based therapies in neurodegenerative disorders such as amyotrophic lateral sclerosis [49–52], Multiple Sclerosis [53], and ataxia [16, 23, 24]. Jones et al. conducted a study where they transplanted BM-MSCs into a mouse model of cerebellar ataxia (Lurcher mutant mouse), ), improved motor function, and increased Purkinje cell numbers in the cerebellum [16]. Only a few clinical trials for cell therapy in patients with ataxia are available, with no severe side effects reported thus far. One such trial involved the transplantation of UC-MSCs in patients with SCA, resulting in symptom reduction, delayed disease progression, and improvements in the BBS and ICARS scores over a 12-month follow-up period [24]. Additionally, a study involving three patients with FRDA demonstrated that four rounds of allogeneic grafting of UC-MSC led to improved motor performance and enhanced quality of life [54].

While our study indicates positive outcomes following cell therapy for patients, we acknowledge the limitations of this research. The sample size was relatively small and did not contain all types of HCA. Also, this study was one arm and did not contain a control group. Short follow-up is another weakness of this study. The absence of imaging or objective neurobiological endpoints (e.g., MRI volumetry, diffusion imaging, or spectroscopy) is a key limitation. Clinical scales such as SARA can be influenced by short-term variability, expectation effects, or learning. Future trials should incorporate structural/functional imaging to better correlate clinical, biological, and anatomical outcomes [55].

## Conclusion

Our findings suggest that intrathecal transplantation of a single dose of BM-MSCs in patients with HCA is a safe and well-tolerated procedure in the short term. Preliminary observations suggest possible clinical stabilization or improvement and modulation of selected inflammatory biomarkers. However, trends observed in SARA scores and biomarker profiles must be interpreted with considerable caution due to the very small sample size, marked disease heterogeneity, lack of a control group, short follow-up duration, lack of adjustment for multiple comparisons, and potential confounding factors. Therefore, no definitive conclusions regarding efficacy can be established from the present study. Rather, these results should be viewed as exploratory and hypothesis-generating, providing a basis for the design of larger, randomized, controlled trials to further evaluate the safety, biological effects, and therapeutic potential of BM-MSC therapy in hereditary cerebellar ataxias.

## Data Availability

All data generated or analyzed during this study are included in this published article.

## Abbreviations

HCA: Hereditary cerebellar ataxias
MSCs: Mesenchymal stem cells
UC-MSCs: Umbilical cord MSC
BM-MSCs: Bone marrow-derived mesenchymal stem cells
SARA: Scale for the Assessment and Rating of Ataxia
CSF: Cerebrospinal fluid
SCA: Spinocerebellar ataxia
FRDA: Friedreich ataxia
FXN: Frataxin gene
GAD: Glutamic acid decarboxylase
GABA: Gamma-aminobutyric acid
